# Host Races of the Cotton Aphid, *Aphis gossypii*, in Asexual Populations from Wild Plants of Taro and Brinjal

**DOI:** 10.1673/031.013.3401

**Published:** 2013-04-18

**Authors:** B.K. Agarwala, Parichita Ray Choudhury

**Affiliations:** Ecology and Biodiversity Laboratories, Department of Zoology, Tripura University, Suryamaninagar, Tripura 799 022, India

**Keywords:** host adaptation, reciprocal-host transfers

## Abstract

Worldwide, several studies have shown that adaptation to different host plants in phytophagous insects can promote speciation. The cotton aphid, *Aphis gossypii* Glover (Homoptera: Aphididae: Aphidini), is a highly polyphagous species, but its populations increase by parthenogenetic reproduction alone in Indian subcontinent. This study showed that genotypes living in wild plants of taro, *Colocasia esculenta* var. *esculenta* (L.) Schott (Alismatales: Araceae), and brinjal, *Solanum torvum* Swartz (Solanales: Solanaceae), behave as distinct host races. Success rates of colonization after reciprocal host transfers were very poor. Clones of *A. gossypii* from wild taro partly survived in the first generation when transferred to wild brinjal, but nymph mortality was 100% in the second generation. In contrast, brinjal clones, when transferred to taro, could not survive even in the first generation. Significant differences between the clones from two host species were also recorded in development time, generation time, fecundity, intrinsic rate of increase, net reproductive rate, and mean relative growth rate. Morphologically, aphids of wild taro clones possessed longer proboscis and fore-femora than the aphids of the brinjal clones. The results showed that *A. gossypii* exists as distinct host races with different abilities of colonizing host plants, and its populations appear to have more potential of sympatic evolution than previously regarded.

## Introduction

Asexual populations of the cotton aphid, *Aphis gossypii* Glover (Homoptera: Aphididae: Aphidini), a worldwide pest in agriculture, horticulture and, greenhouse crops (Agarwala and Ghosh 1985; Blackman and Eastop 1984), show clonal diversity in relation to host plants in several parts of the world (Inaizumi 1981; Moursi et al. 1985; Guldemond et al. 1994; Wool and Hales 1996; Fuller et al. 1999). This aphid species is now considered to consist of distinct genotypes, both holocyclic and anholocyclic, that vary with respect to their ability to reproduce and host preferences on different host plants (Takada and Murakami 1988; Zhang and Zhong 1990; Mokhtar et al. 1993). These varying genotypes imply that the evolutionary potential of *A. gossypii* to adapt to unused plant species might be larger than previously thought, and emphasize the great potential of *A. gossypii* as a major pest species on an increasing number of crops. Available literature suggests that several of the genotypes of *A. gossypii* are adapted to different host plants and might be considered as host races (Jaenike 1981; Diehl and Bush 1984). In the green bug, *Schizaphis graminum*, several biotypes were distinguished on the basis of their performance on different cereals (Bregovoy et al. 1988; Powers et al. 1989; Wilhoit and Mittler 1991), a difference that is supported by RAPD-PCR studies (Lushai et al. 1997). In India, Agarwala and Das (2007) reported hostplant-based morphological, ecological, and esterase variations in *A. gossypii* populations from cotton and chili plants. Although the recorded variations were not found to be unique to respective plant species, that study, along with results of several other earlier studies, as stated above, presented credible evidence that *A. gossypii* shows adaptation to host environments of different plant species across their geographical distribution.

Wild plants of taro, *Colocasia esculenta* var. *esculenta* (L.) Schott (Alismatales: Araceae), and brinjal, *Solanum torvum* Swartz (Solanales: Solanaceae), occur commonly in the moist and hot climate of Tripura (22° 56′ to 24° 32′ N and 91° 10′ to 91° 21′ E) and elsewhere in northeast India (Deb 1981). These plants attract *A. gossypii*, which form small to large colonies on the undersides of leaves and tender shoots of these hosts. In the field, asexual, wingless, and viviparous, morphs (apterae) of *A. gossypii* showed sharp differences in body color and colonization behavior on the two host plants, which possibly provided different food environments to the aphids. It was predicted that *A. gossypii* of wild plants of taro and brinjal might be different races due to the influence of their host environments. Environment mediated host races were earlier described in *A. gossypii* populations from several plants belonging to different families (Guldemond et al. 1994; Carletto et al. 2009). A host race represents a genetically differentiated population of a species that showed preference for a particular host plant, in the case of phytophagous insects. Such a population requires host specialization (Feder 1998; Dres and Mallet 2002). As aphids from taro and brinjal hosts showed essential similarities in morphological attributes of taxonomic importance (including shape, size, lengths, and ratios of: body, antennae, cauda, siphunculi, body hairs, texture of dorsal and ventral surfaces, ultimate rostral segments, and hind tarsi (Eastop 1966; Raychaudhuri 1980)), it was assumed that these populations of *A. gossypii* might represent an intermediate stage along the species continuum, show divergent host selection, and are yet to attain the critical threshold of species separation. As of now, there is no clear guide as to how much genetic isolation or gene flow indicates a species rather than a host race (Berlocher 1999).

In the present study, population parameters comprised of developmental and reproductive fitness, and morphological characteristics, of *A. gossypii* populations from wild plants of taro and brinjal were investigated. The performance of clones of *A. gossypii* originating from wild taro and wild brinjal were also subjected to reciprocal host transfers, and the effect of induction of a new host environment was recorded to test the prediction that they are different races.

## Materials and Method

### Insects

Apterous, parthenogenetic, viviparous aphids of *Aphis gossypii* were collected from taro and brinjal plants found in the wild at five different locations, separated by about 2000 m distance from each other, in and around Agartala, northeast India (23.50° N latitude, 91.25° E longitude). These aphids were used to raise stock cultures, ten each of *A. gossypii* on taro and on brinjal plants under greenhouse conditions (24 ± 1° C temperature and 16:8 L:D photoperiod).

Host plants of the two species in the early vegetative stage were maintained individually in clay or plastic pots, and these were held in water trays on benches illuminated with photo-synthetically active radiation lamps. Individual plants, two from each location, were infected with a single, fourth instar, apterous aphid collected from their respective locations in the fields. These were allowed to grow, reproduce, and increase in number. Aphid cultures on individual potted plants were confined in nylon net cages in segregated locations. This was repeated ten times for each plant species. All aphids produced from a single mother on each of the plants by this practice consisted of the same genotype and thus constituted a clone. Fourth instar aphids produced of the same genotype of a grandmother on a plant species were used in experiments. Individual aphids, chosen randomly from taro and brinjal clones in the greenhouse, were placed on the apical part of the 16–20-day-old pot-grown saplings at the early vegetative stage in a rearing cabinet (temperature: 24 ± 1° C; 65% RH, and 16:8 L:D photoperiod). Thus, several sister clones of the same genetic lineage of the two aphid clones were raised on their two host plant species. Aphid-infested individual plants were individually caged to avoid any contamination during the experiment. Observations were made at frequent intervals until each clone attained its maximum increase in population and then started to decline. At this point, plants were replaced by fresh ones in order to maintain the vigor of the aphid culture. Sister clones were monitored individually several times a day. Alate females were discarded. Aphids from these clones representing two different genotypes from the two host plant species were used to measure differences in their developmental, reproductive, and morphological characters. For determining the mean relative growth rate, parthenogenetic females of aphids from their respective clones were enclosed individually in leaf cages (Blackman 1987) to obtain parthenogenetic descendents. Individual aphids were monitored for weight at birth (< 12 hr) and at the final molt during their development.

### Population parameters

Maximum population size and growth rate were determined for the genotypes of *A. gossypii* from the two host plant species. Twenty replicates were used in the study, ten on each plant species. Maximum population size (*N_t_*) size (*N_t_*) of a clone achieved on a potted plant and the time (*T*) taken to reach the *N* were used to compare any difference in the performance of *A. gossypii* clones on their host plants. Population growth rate (GR), denoting the increase in the number of aphids of a clone per day per plant in the rising phase of population increase, was calculated by the formula

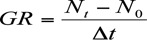

where *N_t_* is the number of aphids recorded at the maximum count of the population on a plant, *N_0_* is the number of aphids initially released on a potted plant, and δ*T* is the difference of time between *N_0_* and *N_t_* (Odum 1971).

The time taken to reach the maximum population size (T) was calculated by the equation T = Σ no. of days to N_t_/ n, where n is the number of observations (Agarwala and Das 2007).

### Developmental and reproductive parameters

Development time (DT), generation time (GT), reproductive duration (RD), and fecundity (F) were recorded for individual aphids of *A. gossypii* of the two different host plant species. In order to record these characteristics, individual third or fourth instar nymphs were placed on a leaf of a potted plant and enclosed in a leaf cage (Blackman 1987) in a temperature-controlled cabinet at 24 ± 1° C. This procedure was repeated ten times for aphids from the two host species. Nymphs were allowed to become apterous adults, to reproduce in the first 24 hours, and then the adults were removed. Only one new-born aphid of an adult was retained, and the rest were removed. Its weight was recorded, and it was allowed to develop to the final molt, at which time it was weighed again and observed for the durations of pre-reproduction, reproduction, and post-reproduction. The number of nymphs born to individual aphids was counted, and all but one aphid were removed. The remaining aphid was allowed to develop in experimental culture. As a result of this procedure, birth weight (BW) of nymphs within 12 hr of laying by a mother aphid, adult weight at the final molt (AW), developmental time from birth of a nymph to its final molt, generation time from the birth of a nymph to the onset of reproduction by this nymph, reproductive duration from the birth of the first nymph to the last nymph by an apterous female, and fecundity were recorded. The time interval in hours from the molting of third instar to the shedding of skin by fourth instar aphids was used to determine the duration of final molt (D*FM*). Molting of third and fourth instars was monitored, and molted skin was removed soon after the measurements were recorded. The time interval in hours from the final molt to the production of the first nymph by an apterous adult aphid was recorded as the D_*1st PG*_ (Bhadra and Agarwala 2010).

Mean relative growth rate, a measure for assessing the performance of different clones of the same species under different environmental conditions (Radford 1967), was determined following the method of Watt and Hales (1996):

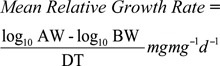

where AW = adult weight in mg, and is expressed as mg increase in weight of aphids born per mg of the mother aphid per day.

The net reproductive rate (*R_0_*), the multiplication rate of an organism per generation, was calculated using the following equation (Krebs 1985):

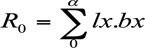

where *l_x_* is the proportion of female aphids surviving, and *bx* is the number of female offspring produced per female during its reproductive time.

The intrinsic rate of increase (*R_max_*), a measure of the rate of increase of a population under controlled conditions, was calculated using the formula:

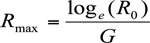

where *G* is the mean length of a generation, determined as under (Dublin and Lotka 1925):

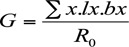

where *x* is the age of female adults.

### Morphological variations

Twenty adults of similar age were individually collected from the clones of two host plant species. These were processed as whole mounted specimens on glass slides for microscopic examination following the method of Raychaudhury (1980). The following different characters of taxonomic importance were measured with an eye-piece micrometer at 400× magnification using a light microscope: (1) length of body (BL), (2) maximum width of body (MW), (3) length of antenna (ANT), (4) length of antennal segment III (ANT III), (5) length of antennal segment VI (ANT VI), (6) length of proboscis (PROB), (7) length of ultimate rostral segments (URS), (8) length of fore femur (FEM), (9) length of siphunculus (SIPH), and (10) length of cauda (CAU).

### Host transfer experiments

Aphids of clones from the taro and brinjal hosts were subjected to reciprocal host transfer to record the colonization success in a new food environment. Two experiments were set up using parental clones of *A. gossypii* from the two host plant species. In the first treatment, *A. gossypii* aphids were transferred individually from the wild taro field host to the laboratory host, wild brinjal. In the second treatment, *A. gossypii* aphids were transferred from the wild brinjal field host to wild taro as the laboratory host. Individual nymphs, 0–12 hr old, were released at the apical-most part of potted plants of 12–16 days old of field hosts (control) and laboratory hosts (treatments). These aphids were allowed to settle and produce nymphs for the first generation. If successful, a second and a third generation were produced. Ten replicates were used in each experiment to record the success rate of survival and reproduction by apterous, viviparous aphids of a host plant, leading to the establishment of colony. Aphids that either failed to develop to the adult stage in the first generation or failed to produce second generation or third generation were considered to be unsuccessful.

### Data analysis

Data of the third generation aphids, wherever available, were used to compare the results of population, developmental, reproduction, and morphological parameters. Third generation aphids were used in order to allow the aphids sufficient time for acclimatization to the laboratory rearing conditions. All microscopic measurements were converted to mm using a stage micrometer. All weights in this study were taken in a Mettler microbalance (www.met.com) sensitive to 2 µg. Each of the population, developmental, reproduction, and morphological parameters that were measured from the wingless aphids from different *A. gossypii* clones met the criteria of normality and equal variance, and these were compared using Student's *t*-test. A comparison of frequency of success and failure in colonization by *A. gossypii* aphids on different host plant species in the host transfer experiments was tested by chi-squared test. Origin 7 (www.originlab.com) was used for the analysis of data.

## Results

### Body color and colonization pattern

**Figure 1.**
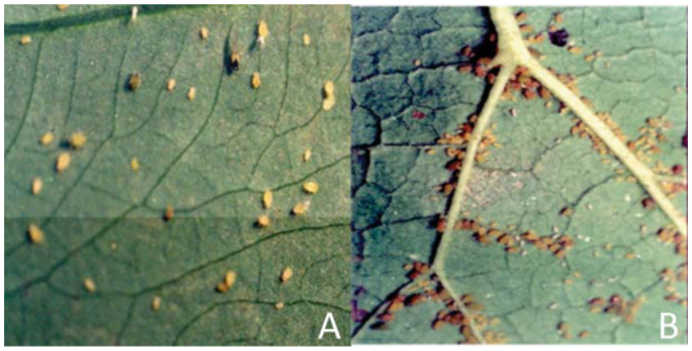
*Aphis gossypii* colony on taro and brinjal leaves: A: pale-yellow aphids forming dispersed colony in laminar part of taro leaf; B: dark yellow aphids forming gregarious colony along veins of in brinjal leaf. High quality figures are available online.

*A. gossypii* from taro were pale yellow and occurred all over the laminar surface, as well as along the veins on the ventral surface of leaves. Several independent discrete colonies simultaneously occurred on a leaf. Heavily infested leaves show dispersed or loose aggregate of aphids without any continuity between the colonies (Figure 1A). Aphids of brinjal hosts are bright yellow in color. Colonization mostly occurred around the primary vein or bases of secondary veins on ventral surface of leaves. Colony did not spread to laminar area. Heavily infested leaves show dense aggregation of aphids in unbroken linear arrangement in primary and secondary veins (Figure 1B).

**Table 1.**
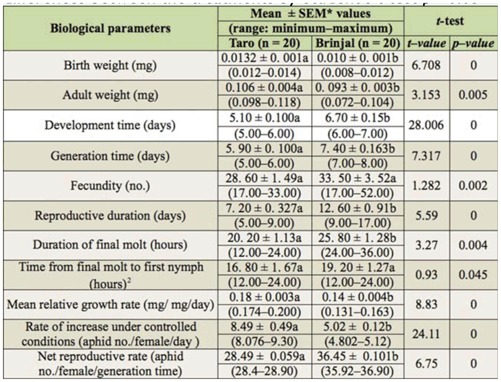
Mean values of biological parameters studied in *Aphis gossypii* clones from wild species of taro and brinjal host plants. Different letters with mean values in a row indicate significant differences between the treatments by Student's t test *p* <0.05

### Population parameters

**Figure 2.**
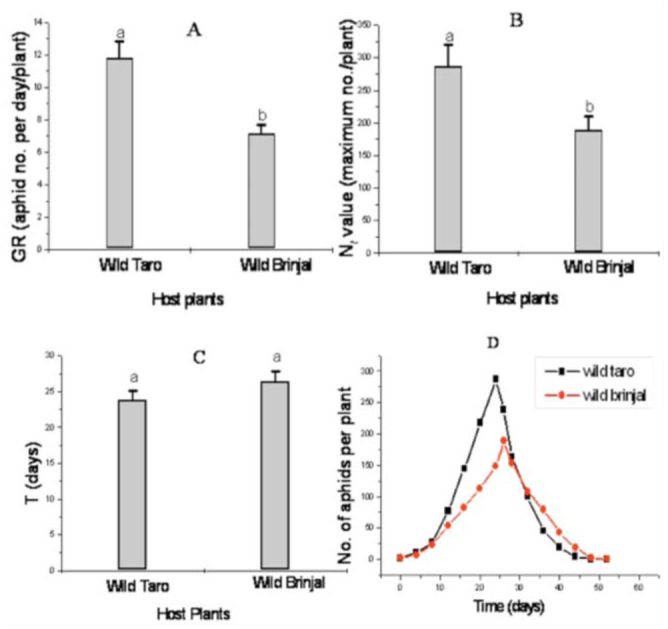
A: mean values of growth rate (GR); B: maximum population size (N*t*); C: time to attain N*t* (T); and D: population trend of *Aphis gossypii* determined on potted wild plants of taro and brinjal. Error bars accompanying means represent standard errors of means; different letters accompanying error bars denote significant difference between the means by Student's *t*-test at *p* < 0.05. High quality figures are available online.

Clones of *A. gossypii* from wild taro and brinjal plants showed significant differences in growth rates (Figure 2A) and maximum population size attained (Figure 2B) on their respective host plants. The average growth rate of *A. gossypii* clones from brinjal was significantly slower in comparison to that of clones from taro (mean ± SEM: brinjal = 7.12 ± 0.55 aphids/day/plant; taro = 11.8 ± 1.04 aphids/day/plant; *t*-value = 3.39, df = 18, *p* = 0.001; Figure 2A). Maximum population size of *A. gossypii* clones on taro plants was 1.5 times higher than that of clones on brinjal plants (mean ± SEM: taro = 287.3 ± 13.22 aphids/plant; brinjal = 189.1 ± 12.18 aphids/plant; *t* = 0.6613, df = 18, *p* = 0.044; Figure 2B). However, the time taken by the clones to achieve the maximum population size on their respective host plants did not show significant difference (*t* = 1.29, df = 18, *p* = 0.214; Figure 2C). Thus, *A. gossypii* clones on taro formed bigger colonies in comparison to clones on brinjal during the same time (Figure 2D).

### Developmental and reproduction parameters

Apterous aphids of *A. gossypii* clones from wild plants of taro and brinjal, respectively, showed significant differences in size of aphids at birth and at final moult, development time, generation time, reproductive time, durations of final moult, mean relative growth rate, intrinsic rate of increase, and net reproductive rate (Table 1). Aphid size at birth and at final moult of taro clones were 1.30 times and 1.13 times bigger, respectively, in comparison to aphids reared on brinjal host. Development time and generation time, however, of aphids of brinjal clones were longer by 24% and 19%, respectively, in comparison to that of taro clones. The mean relative growth rates of brinjal and taro clones also showed a significant difference (*t* = 8.83, df = 18, *p* < 0.01). Mean relative growth rate was higher (> 1.27 times) in taro clones in comparison to the brinjal clones. The difference in fecundity between the aphids of clones from two host species was not significant (*t* = 1.28, df = 18, *p* = 0.216). Reproductive time, however, was significantly longer in aphids of brinjal clones (1.75 times) in comparison to that from the taro clones (*t* = 5.59, df = 18, *p* = 0.01). Aphids of taro clones achieved a significantly higher rate of increase than the aphids of brinjal clones (*t* = 24.11, df = 18, *p* = 0.01). The net reproductive rate of brinjal clones was recorded to be higher by about 1.28 times in comparison to the aphids of taro hosts. Average time (hours) taken by aphids in the third moult to become the final moult was found to be significantly higher in aphids reared on brinjal than those reared on the taro hosts. However, the mean time taken to produce the first progeny by females on brinjal and taro hosts was found to be nearly the same (*t* = 0.93, df= 18, p < 0.045 (Table 1).

### Morphological parameters

**Figure 3.**
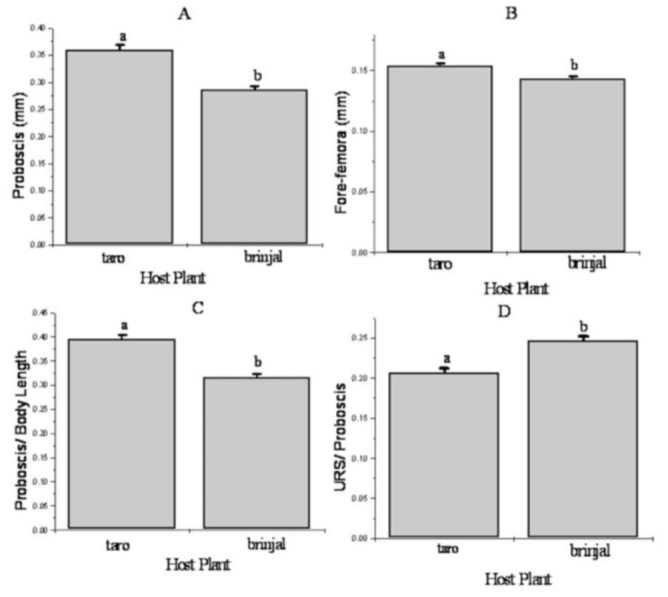
Variations in mean (mm) values of morphometry recorded in apterous aphids of *Aphis gossypii* clones of taro and brinjal hosts: A: proboscis, B: fore-femur, C: ratio of proboscis to body length, D: and ratio of ultimate rostral segments (URS) to proboscis. Different letters accompanying the standard error bars indicate significant difference between them by Student *t*-test at *p* < 0.05. High quality figures are available online.

In general, aphids of *A. gossypii* clones from the two host plant species showed similarities in the diagnostic characters of this species. However, aphids of the brinjal clones were larger and possessed longer proboscis (Figure 3A, taro = 360 ± 0.009; brinjal = 287 ± 0.006) and shorter fore-femora (Figure 3B, taro = 154 ± 0.002; brinjal = 147 ± 0.005) in comparison to that of the taro clones (Figure 3A). The ratios of proboscis to body length and ultimate rostral segments to body length showed distinguishable variations between the two aphid genotypes (Figure 3C, 3D).

**Figure 4.**
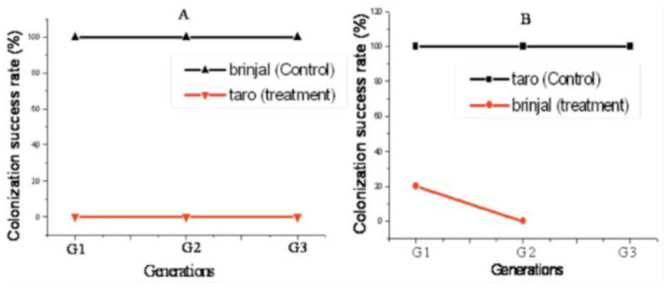
Success of colonization of *Aphis gossypii* through generations on their field hosts (control) and across host plants. A: treatment 1: *A. gossypii* of brinjal transferred to laboratory host taro. B: treatment II: *A. gossypii* of taro transferred to laboratory host brinjal. High quality figures are available online.

### Host transfer experiments

Aphids of *A. gossypii* clones from wild species of brinjal plants all died when transferred to wild taro plants (Figure 4A). Likewise, aphid clones from wild taro hosts could not survive when transferred to wild brinjal plants (Figure 4B).

## Discussion

Recent morphological, biochemical, and host plant preference studies have shown that a number of aphid species, notably polyphagous species, consist of genetically different forms, i.e., host races, or even appear to represent complexes of several separate species (Inaizumi 1980; Wool et al. 1995; Fuller et al. 1999). Guldemond et al. (1994) recorded significant differences in the biological performance of *A. gossypii* on cotton, cucumber, and okra.

In the present study, *A. gossypii* populations on wild species of taro and brinjal host plants showed profound differences in most of the characters studied. The responses of aphids to a new host environment were found to represent host specialization in the aphid-host relationship. *A. gossypii* on taro showed longer proboscis and longer fore-femora than the *A. gossypii* on brinjal. Significant differences were also recorded in biological attributes, such as adult weight, development time, generation time, fecundity, reproductive duration, intrinsic rate of increase, net reproductive rate, and mean relative growth rate, between *A. gossypii* clones from taro and brinjal. Reciprocal transfer of *A. gossypii* populations from taro to brinjal and vice-versa was not possible, even in the first generation. A success rate of zero for *A. gossypii* clones on unfamiliar plants of different genera suggested the inability of the respective laboratory clones to accept a new host environment. The results suggest that clonal populations of *A. gossypii* perform best on their respective host plants. *A. gossypii* populations exhibit host plant specialization within a narrow range of host selection. As a consequence, *A. gossypii* can be considered to represent a genetically heterogeneous species infesting different host plants at different rates, i.e., *A. gossypii* consists of different host races according to the definition of Jaenike (1981) and Diehl and Bush (1984). This specialization implies that no or little infestation will occur of *A. gossypii* populations from taro to brinjal and vice versa.

In Japan, China, and the USA, some populations of *A. gossypii* showed cyclical parthenogenesis consisting of one sexual generation followed by several asexual generations (Inaizumi 1981; Ebert and Cart-wright 1997), and these aphids performed better on cotton, cucurbits, and chrysanthemum than on other host plants, with wide variations in their colonization success and rate of increase. These host-based relations have been attributed to a genetic component due to variations in sexual populations from different plants (Guldemond et al. 1994; Wool et al. 1995). Given that there has been no reported occurrence of sexual reproduction in *A. gossypii* in India, the chief factor that might be contributing to the observed variability in *A. gossypii* populations from different plant species could be the host plant specialization. In this scheme, asexual, viviparous aphids of *A. gossypii* undergo constant pressure of host selection in patchy habitats of mixed vegetation, and the choice of host selection could be chiefly determined by the proximate causes of interactions between the aphid and the host environment (Jaenike 1990; Dixon 1998; Powell et al. 2006). The host environments of taro and brinjal plants are very different (Flick Jr. et al. 1978; Egbe and Rickard 1990; Estaben et al. 1992; Onwueme 1999), yet they offer the choice to essentially morphologically similar *A. gossypii* aphids to select these hosts. The results of this study have shown that aphid-host plant interactions in natural *A. gossypii* populations have produced different fitness on different hosts, fitness being manifested by the ability to reproduce in response to preferred host cues and showing different rates of increase. These different *A. gossypii* forms are evidently specialized genotypes. The results also imply that the effects of aphid-host plant interactions produce plasticity in phenotypes, showing different reaction norms on different host species (Agarwala 2007). Using random amplified polymorphic DNA markers, Vanlerberghe-Masutti and Chavigny (1998) showed that populations of *Aphis gossypii* collected on plants of the same family were multi-clonal. Carletto et al. (2009) identified five host races of *A. gossypii* dominated by asexual clones from as many plant species based on genetic diversity using microsatellites analysis. Despite several records of host specialization in *A. gossypii* from different parts of the world, current data do not provide unambiguous genetic discontinuity between different populations on different host plants for these to be considered as distinct species (Brevault et al. 2008; Komazaki and Toda 2008).

Similar mechanism of plasticity has been reported in oligophagous *Lipaphis pseudobrassicae* (Kaltenbach). Populations of this species from *Rorippa* host are found to be genetically different from the populations that feed on sarson mustard, *Brassica campestris* L., and rai mustard, *Brassica juncea* (L.) Czern and Coss (Agarwala et al. 2009). Although it is not clearly understood as to how host plant selection and performance are genetically related, several biotic and abiotic factors can contribute to their relationship (Via 1991; Caillaud and Via 2000; Egas and Sabelis 2001). The available results of speciation in phytophagous insects are based on the concepts of plant preference and performance on preferred host plants (Powell et al. 2006).

Most of the observed differences in ecological and biological attributes and morphometrics of the *A. gossypii* forms in this study suggested the occurrence of underlying genotypic differentiation in aphid populations within an aphid species. In absence of gene flow and genetic recombinations in these populations, obligate parthenogenesis has the advantage of fast reproduction rates, and the fast reproduction rates could amplify the effects of trivial life history differences so that frequencies of genotypes showing small differences in a trait may differ considerably after several generations (Mackenzie and Guldemond 1994). It could be assumed that several distinct genotypes or host races of *A. gossypii* might be occurring in the hitherto un-explored mountainous regions of northeast India, which is the confluence of Malayan, Mayanmar, and Chinese biogeography.

## References

[bibr01] Agarwala BK (2007). Phenotypic plasticity in aphids (Homoptera: Insecta): Component of variation and causative factors.. *Current Science*.

[bibr02] Agarwala BK, Das K (2007). Host-plant based morphological, ecological and esterase variations in *Aphis gossypii* Glover populations (Homoptera Aphididae).. *Entomon*.

[bibr03] Agarwala BK, Das K, Raychoudhury P (2009). Morphological, ecological and biological variations in the mustard aphid, *Lipaphis pseudobrassicae* (Kaltenbach) (Hemiptera: Aphididae) from different host plants.. *Journal of Asia Pacific Entomology*.

[bibr04] Agarwala BK, Ghosh MR (1985). Biogeographical considerations of Indian Aphididae (Homoptera).. *Insecta Matsumurrana*.

[bibr05] Berlochar SH (1999). Host race or species? Allozyme characterization of the ‘flowering dogwood fly’, a member of the *Rhagoletis pomonella* complex.. *Heredity*.

[bibr06] Bhadra P, Agarwala BK (2010). A comparison of fitness charaters of two host plantbased congeneric species of the banana aphid, *Pentalonia nigronervosa* and *P. caladii*.. *Journal of Insect Science*.

[bibr07] Blackman RL, Minks AK, Harrewijn P (1987). Rearing and handling aphids.. *Aphids: Their biology, Natural Enemies and Control*, volume 2B..

[bibr08] Blackman RL, Eastop VF (1984). *Aphids on the World's Crop: An identification Guide*..

[bibr09] Bregovoy VH, Starks KL, Janardan KG (1988). Fecundity characteristics of the green biotypes C and E cultured on different host plants.. *Environmental Entomology*.

[bibr10] Brevault T, Carletto J, Linderme D, Vanlerberghe-Masutti F (2008). Genetic diversity of the cotton aphid *Aphis gossypii* in the unstable environment of a cotton growing area.. *Agricultural and Forest Entomology*.

[bibr11] Caillaud MC, Via S (2000). Specialized feeding behavior influences both ecological specialization and assortative mating in sympatic host races of pea aphids.. *American Naturalist*.

[bibr12] Carletto J, Lombart E, Chavigny P, Brevault T, Lapchin L, Vanlerberghe-Masutti F (2009). Ecological specialization of the aphid *Aphis gossypii* Glover on cultivated host plants.. *Molecular Ecology*.

[bibr13] Deb DB (1981). *The flora of Tripura State*, volume 1..

[bibr14] Diehl SR, Bush GL (1984). An evolutionary and applied perspective in insect biotypes.. *Annual Review of Entomology*.

[bibr15] Dixon AFG (1998). *Aphid Ecology*..

[bibr16] Dres M, Mallet J (2002). Host races in plant feeding insects and their importance in sympatic speciation.. *Philosophical Transactions of the Royal Society of London*.

[bibr17] Dublin LL, Lotka AJ (1925). On the true rate of natural increase as exemplified by the populations of United States, 1920.. *Journal of American Statistics*.

[bibr18] Eastop VF (1966). A taxonomic study of Australian Aphidoidea (Homoptera).. *Australian Journal of Zoology*.

[bibr19] Ebert TA, Cartwright B (1997). Biology and ecology of *Aphis gossypii* Glover (Homoptera: Aphididae).. *Southwest Entomology*.

[bibr20] Egas M, Sabelis MW (2001). Adaptive learning of host preference in a herbivorous arthropod.. *Ecology Letters*.

[bibr21] Egbe TA, Rickard JE (1990). Evaluation of the chemical composition of fresh and stored edible aroids.. *Journal of the Science of Food and Agriculture*.

[bibr22] Estaben RM, Molla EM, Robredo L, Andreu FJL (1992). Changes in the chemical composition of egg plant fruits during development and ripening.. *Journal of Agricultural and Food Chemistry*.

[bibr23] Feder JL, Howard DJ, Berlochar SH (1998). The apple maggot fly, *Rhagoletis pomonella*: flies in the face of conventional wisdom about speciation?. *Endless forms: Species and Speciation*..

[bibr24] Flick GJ, Burnette FS, Aung LH, Robert RL, Angelo AJS (1978). Chemical composition and biochemical properties of mirlitons (*Sechium edule*) and purple, green, and white eggplants (*Solanum melongena*).. *Journal of Agricultural and Food Chemistry*.

[bibr25] Fuller SJ, Chavigny P, Lapchin L, Vanlerberghe-Masutti F (1999). Variation in clonal diversity in greenhouse infestations of the aphid, *Aphis gossypii* Glover in Southern France.. *Molecular Ecology*.

[bibr26] Guldemond JA, Tiggers WT, Vrijer PWF (1994). Host races of *A. gossypii* Glover on cucumber and chrysanthemum.. *Environmental Entomology*.

[bibr27] Inaizumi M (1980). Studies on the life cycle and polymorphism of *Aphis gossypii* Glover (Homoptera: Aphididae).. *Special Bulletin of the College of Agriculture, Utsunomia University*.

[bibr28] Inaizumi M (1981). Life cycle of *Aphis gossypii* (Homoptera, Aphididae) with special reference to biotype differentiation on various host plants.. *Kontyu*.

[bibr29] Jaenike J (1981). Criteria for ascertaining the existence of host races.. *American Naturalist*.

[bibr30] Jaenike J (1990). Host specialization in phytophagous insects.. *Annual Review of Ecology and Systematics*.

[bibr31] Komazaki S, Toda S (2008). Difference in host preference, life cycle pattern, and insecticide susceptibility among *Aphis gossypii* clones and genetic relationships inferred from internal transcribed spacer 2 sequences of rDNA.. *Annals of the Entomological Society of America*.

[bibr32] Krebs CJ (1985). *The Ecology: The Experimental Analysis of Distribution and Abundance*.

[bibr33] Lushai G, Loxdale HD, Brookes CP, Mende NV, Harrington R, Hardie J (1997). Genotypic variation among different phenotypes within aphid clones.. *Proceedings of the Royal Society of London, Series B*..

[bibr34] Mackenzie A, Guldemond JA, Leather SR, Watt AD, NJ Mills, Walters KFA (1994). Sympatic speciation in aphids II. Host race formation in the face of gene flow.. *Individuals, Populations and Patterns in Ecology*..

[bibr35] Mokhtar AM, Polgar L, Lucas S, Kindlmann P, Dixon AFG (1993). Morphological characteristics and host preference of anoholocyclic forms of *Aphis gossypii* Glover (Homoptera, Aphididae) originated from Egypt, Hungary and Sultanate of Oman.. *Critical issues in aphid biology*..

[bibr36] Moursi KS, Donia AA, Mesbah HA, Haroun NS (1985). Comparative biological studies of *Aphis gossypii* Glover on different host plants.. *Annals of Agricultural Science*.

[bibr37] Odum EP (1971). *Fundamentals of Ecology*.

[bibr38] Onwueme I (1999). Composition of taro corm and leaf.. *Taro Cultivation in Asia and the Pacific*..

[bibr39] Powell G, Tosh CR, Hardie J (2006). Host plant selection by aphids: behavioral, evolutionary and applied perspectives.. *Annual Review of Entomology*.

[bibr40] Powers TO, Jensen SG, Kindler D, Stryker CJ, Sandall LJ (1989). Mitochondrial DNA divergence among greenbug (Homoptera: Aphididae) biotypes.. *Annals of the Entomological Society of America*.

[bibr41] Radford PJ (1967). Description of some new or little known species of *Aphis* of Japan with a key to species.. *Transactions of the American Entomological Society*.

[bibr42] Raychaudhuri DN (1980). *Aphids of North East India and Bhutan*..

[bibr43] Takada H, Murakami V (1988). Esterase variation and insecticide resistance in Japanese *Aphis gossypii*.. *Entomologia Experimentalis et Applicata*.

[bibr44] Vanlerberghe-Masutti F, Chavigny O (1998). Host-based genetic differentiation in the aphid species *Aphis gossypii* Glover, evidence from RAPD fingerprints.. *Molecular Ecology*.

[bibr45] Via S (1991). The genetic structure of host plant adaptation in a spatial patchwork demographic variability among reciprocally transplanted pea aphid clones.. *Evolution*.

[bibr46] Watt M, Hales DF (1996). Dwarf phenotype of the cotton aphid, *Aphis gossypii* Glover (Hemiptera Aphididae).. *Australian Journal of Entomology*.

[bibr47] Wool D, Hales DF (1996). Components of variation of morphological characters in Australian *Aphis gossypii*: host-plant effects predominate.. *Entomologia Experimentalis et Applicata*.

[bibr48] Wool D, Hales DF, Sunnucks P (1995). Host plant relationships of *Aphis gossypii* Glover (Hemiptera: Aphididae) in Australia.. *Journal of Australian Entomology*.

[bibr49] Wilhoit LR, Mittler TE (1991). Biotypes and clonal variations in greenbug (Homoptera: Aphididae) populations from a locality in California.. *Environmental Entomology*.

[bibr50] Zhang GX, Zhong TS, Cambell RK, Eikenbary RD (1990). Experimental studies on some aphid life cycles and the hybridization of two sibling species.. *Aphid-plant genotype interactions*..

